# PRECARITY AND AGING: THE SOCIAL CONSTRUCTION OF RISK IN LATER LIFE

**DOI:** 10.1093/geroni/igz038.2182

**Published:** 2019-11-08

**Authors:** Christopher Phillipson

**Affiliations:** The University of Manchester, Manchester, United Kingdom

## Abstract

This paper examines the growth of precarious lives in the context of policies which have marginalised the welfare state and priorities of social inclusion/security. Blackburn observes that ageing societies ‘requires new welfare principles not their repudiation’. However, the reality has been the erosion of the social solidarity which gave the welfare state legitimacy. In Europe, one set of welfare and related institutions is being abandoned, replaced by precarious arrangements -- extended working lives, privatization of pensions, and greater demand for women especially) for unpaid work. This paper 1) draws upon perspectives from political economy and sociology to examine precarity, 2) considers ‘precarious ageing’ as a competing or complementary view to theories of ‘active’ and ‘successful ageing’ third, examines specific dimensions of precarity (work, resources, the environment, frailty), assessing prospects for an index of precarity and aging; finally, considers the public policy implications of the growth of old-age precarity .

